# Heme oxygenase-1-transduced bone marrow mesenchymal stem cells in reducing acute rejection and improving small bowel transplantation outcomes in rats

**DOI:** 10.1186/s13287-016-0427-8

**Published:** 2016-11-20

**Authors:** Yang Yang, Hong Li Song, Wen Zhang, Ben Juan Wu, Nan Nan Fu, Chong Dong, Zhong Yang Shen

**Affiliations:** 1 Department of Organ Transplantation, Tianjin First Central Hospital, 24# Fukang Road, Nankai District, Tianjin, 300192 People’s Republic of China; 2 Tianjin Key Laboratory of Organ Transplantation, 24# Fukang Road, Nankai District, Tianjin, 300192 People’s Republic of China

**Keywords:** Acute cellular rejection, Bone marrow mesenchymal stem cells, Heme oxygenase-1, Small bowel transplantation

## Abstract

**Background:**

We determined whether bone marrow mesenchymal stem cells (BMMSCs) transduced with heme oxygenase-1 (HO-1), a cytoprotective and immune-protective factor, could improve outcomes for small bowel transplantation (SBTx) in rats.

**Methods:**

We performed heterotopic SBTx from Brown Norway rats to Lewis rats, before infusing Ad/HO-1-transduced BMMSCs (Ad/HO-1/BMMSCs) through the superficial dorsal veins of the penis. Respective infusions with Ad/BMMSCs, BMMSCs, and normal saline served as controls. The animals were sacrificed after 1, 5, 7, or 10 days. At each time point, we measured small bowel histology and apoptosis, HO-1 protein and mRNA expression, natural killer (NK) cell activity, cytokine concentrations in serum and intestinal graft, and levels of regulatory T (Treg) cells.

**Results:**

The saline-treated control group showed aggravated acute cellular rejection over time, with mucosal destruction, increased apoptosis, NK cell activation, and upregulation of proinflammatory and immune-related mediators. Both the Ad/BMMSC-treated group and the BMMSC-treated group exhibited attenuated acute cellular rejection at an early stage, but the effects receded 7 days after transplantation. Strikingly, the Ad/HO-1/BMMSC-treated group demonstrated significantly attenuated acute cellular rejection, reduced apoptosis and NK cell activity, and suppressed concentrations of inflammation and immune-related cytokines, and upregulated expression of anti-inflammatory cytokine mediators and increased Treg cell levels.

**Conclusion:**

Our data suggest that Ad/HO-1-transduced BMMSCs have a reinforced effect on reducing acute rejection and protecting the outcome of SBTx in rats.

## Background

Nowadays, small bowel transplantation (SBTx) is the sole therapeutic option for patients with irreversible intestinal failure. However, due to the difficulties associated with the allogeneic immune response, long-term curative effects of SBTx are not comparable with other organ transplantations. Although graft rejection rates for isolated intestinal transplants have decreased from 80% to 30–40% at experienced transplant centers, rejection remains the leading cause of graft failure or loss, and subsequently the main obstacle for further development of SBTx [1–3]. Moreover, administration of immunosuppressive agents to control rejection often causes unfavorable side effects [4].

Mesenchymal stem cells (MSCs) are multipotent nonhematopoietic progenitor cells of stromal origin that can be isolated from the bone marrow or other tissues (e.g., cord blood or adipose tissue) [5] and differentiate into mesoderm-originated cells (osteoblasts, chondrocytes, adipocytes, cardiocytes, etc.), ectoderm-originated cells (nerve cells), and endoderm-originated cells (hepatic oval cells) [6–10]. MSCs have the ability of homing to target tissue. Many studies referring to MSC tracing have demonstrated that intravenously engrafted MSCs are mostly intercepted by microvessels of the lung, heart, liver, kidney, etc., as well as homing to inflammatory and injured areas [11–14]. Importantly, MSCs have been shown to have regenerative, anti-inflammatory, and immunomodulatory capabilities [15–17]. Many experiments and early clinical trials have indicated that MSCs can block the rejection or even induce immune tolerance following transplantation [18, 19]. However, the therapeutic effects of MSCs in transplantation are largely restricted by the limited survival rate in vivo [20]. Reports state that there is an obvious reduction in MSCs 4 days after engraftment, and the survival rate for more than 1 week is less than 1% because of ischemia, anoxia, and the inflammation response [21–25]. Outcomes have the potential to be improved if MSCs are accompanied with specific genetically engineered vectors designed to improve the MSC survival rate.

Heme oxygenase (HO) is the rate-limiting enzyme in the degradation of heme to biliverdin, and subsequently to bilirubin [26]. HO-1, an inducible isoform of HO, is a potent cytoprotective enzyme shown to exhibit anti-inflammatory properties, anti-ischemia–reperfusion injuries, and anti-apoptotic properties [27–29]. In addition, HO-1 has been described as an immunosuppressive molecule and proven to exhibit immunosuppressive effects in adult rat and human MSCs [30, 31]. Importantly, HO-1 represents an endogenous defensive system in organ transplantation and prolonged graft survival [29]. In rodent models, studies have implicated that enhanced HO-1 expression attenuates transplant rejection [32]. Therefore, in this study, we transduced bone marrow MSCs (BMMSCs) with an adenoviral vector carrying the heme oxygenase gene (Ad/HO-1) and infused them into rats after SBTx in order to investigate whether HO-1 gene infection improves the MSC survival rate in vivo and whether Ad/HO-1-transduced MSCs (Ad/HO-1/MSCs) play an enhanced effect in improving outcomes of SBTx in rats.

## Methods

### Animals and ethics

Inbred male Brown Norway (BN) rats (RT-1^n^) and male Lewis rats (RT-1^l^) were purchased from Vital River Company (Beijing, China). Adult BN rats (180–200 g) and Lewis rats (200–220 g) were used as donors and recipients, respectively. Immature Lewis rats (80–100 g) (*n* = 25) were used as a source of BMMSCs. A total of 120 donors (BN rats) and 120 recipients (Lewis rats) were used for SBTx. Of these, 20 recipients (five each in the four groups) were used for survival rate analysis after SBTx. The other 100 recipients were used for sample collection following SBTx (naïve, *n* = 5; 1 day, *n* = 5; 5 days, *n* = 5; 7 days, *n* = 5; 10 days, *n* = 5 in each of the four groups). All animals were treated humanely and housed individually in standard animal facilities, and maintained on a 12:12-h light/dark cycle in a temperature-controlled room (25 °C). Animals were monitored regularly and fed adaptively for at least 3 days before surgery. Rat chow and tap water were provided *ad libitum* until animals were fasted 24 h prior to surgery. This experiment was carried out under strict accordance with the Guide for the Care and Use of Laboratory Animals published by the National Institutes of Health (NIH Publication 86–23, revised 1985), and all protocols were approved by the Animal Care and Research Committee of Tianjin First Central Hospital, Tianjin, China (Permit Number: E20140525-001A). All surgeries and sacrifices were performed under chloral hydrate anesthesia. Every effort was made to minimize animal suffering.

### Preparation and identification of Ad/HO-1/BMMSCs

BMMSC isolation, culture, and characterization techniques were as described previously by our group [33]. Briefly, BMMSCs were isolated from the femur and tibia of male Lewis rats (80–100 g) and cultured in a cell culture incubator. The culture medium was changed and the cells were consistently passaged when 80% confluence was achieved. Following the third passage, BMMSCs were collected for infusion or transduced with adenovirus. Transduction was performed when the third-passage BMMSCs reached 70–80% confluence. Briefly, culture media were renewed and Ad/HO-1 (Jikai, Shanghai, China) was added at a multiplicity of infection (MOI) of 10. The media containing Ad/HO-1 was replaced after 6–8 h of culture. Ad/HO-1/BMMSCs were trypsinized, washed, centrifuged, and resuspended at 1 × 10^7^ cells/ml in Dulbecco’s Modified Eagle’s medium (DMEM) for infusion after 48–72 h of culture. Molecular biological features of Ad/HO-1/BMMSCs were assessed by osteogenic differentiation and adipogenic differentiation abilities in vitro, as well as phenotype identification by flow cytometry (FACSCalibur; BD, Alaska, MN, USA), and stained with antibodies against CD29, CD34, CD45, CD90, RT1A, and RT1B (Biolegend, San Diego, CA, USA).

### Detection of HO-1 expression in Ad/HO-1/BMMSCs

Immunocytochemical staining and western blot analysis were performed to determine whether the HO-1 gene could be overexpressed successfully in BMMSCs transduced with Ad/HO-1. Briefly, for immunohistochemical staining, Ad/HO-1/BMMSCs were stained with a mouse anti-rat HO-1 antibody (Abcam, USA) and TRITC-conjugated goat anti-mouse IgG antibody. The cell nuclei were stained with 4,6′-diamidino-2-phenylindole dihydrochloride (DAPI). For western blot analysis, total protein was released from cells for gel electrophoresis, and then the separated proteins were transblotted from gel to membrane and incubated with polyclonal rabbit anti-rat HO-1 antibody (Stressgen Biotechnologies, Victoria, BC, Canada) and goat anti-rabbit IgG second antibody, respectively. Reactive protein was exposed under the ECL chemiluminescence system.

### Surgical procedures and experimental protocol

Both the donor and recipient rats were randomly assigned to an experimental group and three control groups. Heterotopic SBTx was performed allogeneically from BN (RT1^n^) rats to Lewis (RT1^l^) rats as described in our previous study [34]. For the Ad/HO-1/BMMSC treatment group, recipient rats were treated with 1 × 10^7^ Ad/HO-1/BMMSCs through the superficial dorsal veins of the penis immediately after surgery. Control rats were treated with 1 × 10^7^ Ad/BMMSCs or 1 × 10^7^ BMMSCs. Isopycnic saline infusions served as the allogeneic control (Allo). The animals were monitored after surgery three times per day at least and the weight loss was recorded. The animals were euthanized via an intraperitoneal injection containing excessive chloral hydrate due to pain and suffering experienced from surgical complications and acute rejection. A total of five animals per group were sacrificed 1, 5, 7, and 10 days after SBTx under chloral hydrate anesthesia.

### Survival rate of the recipients

We observed the survival rate and quality of life in five recipient rats per group. The survival rates of the recipients were compared between groups by Kaplan–Meier analysis, and a log-rank test was used to identify any significant differences between the groups. Differences were considered statistically significant when *P* ≤ 0.05. Data are presented as the mean ± standard deviation (SD).

### Clinical manifestation and weight loss of the recipients

The recipients were observed carefully following surgery and graded based on their clinical manifestations, including grooming, mental state, activity, and response to stimulation (0 was scored for normality, 1, 2, and 3 were scored for mild, moderate, and severe change, respectively). Body weight changes were precisely recorded at each time point.

### Western blot analysis of intestine HO-1

Total cell proteins from intestinal grafts were released using Complete Lysis M reagent (Roche, Germany) according to the manufacturer’s instructions. Then, 50 μg protein samples were boiled for 5 min in loading buffer containing 4% sodium dodecyl sulfate (SDS), 20% glycerol, and bromophenol blue. Proteins were separated by 10% and 8% sodium dodecyl sulfate-polyacryl-amide gel electrophoresis (SDS-PAGE) and transblotted onto a polyvinylidene fluoride membrane (Roche, Germany). Nonspecific reactivity was blocked using 5% nonfat dry milk in TBST (10 mmol/L Tris–HCl (pH 7.5), 150 mmol/L NaCl, 0.05% Tween-20) for 1 h at room temperature. The membrane was then incubated with polyclonal rabbit anti-rat HO-1 antibody (Stressgen Biotechnologies) or β-actin (Sigma, USA) at 4 °C overnight. After three washes with TBST, membranes were incubated with horseradish peroxidase-conjugated goat anti-rabbit IgG antibody for 2 h at room temperature. Reactive protein was detected using the ECL chemiluminescence system.

### Detection of intestinal HO-1 mRNA by real-time PCR

To assess the expression of HO-1 by real-time PCR, total RNA was extracted from intestine graft with TRIzol (TakaRa, Japan). RNA (1 mg) was taken for reverse transcription with a reaction volume of 30 ml. PCR was used for HO-1 amplification and β-actin was used as an internal reference. Initial starting concentrations of cDNA were determined by arbitrary units, and the ratio of HO-1/β-actin was used to normalize the different samples. Oligonucleotide primers (Yingjun Biosciences, Shanghai, China) for HO-1 (forward, 5′-TCC CAG ACA CCG CTC CTG CGA-3′; reverse, 5′-GGA TTT GGG GCT GCT GGT TTC-3′) and β-actin (forward, 5′-ATT GCT GAC AGG ATG CAG AAG-3′; reverse, 5′-AGA GCC ACC AAT CCA CAC AGA-3′) were used. PCR conditions included a primary denaturation time of 5 min at 94 °C, followed by 33 cycles of 30 s at 94 °C, 30 s at 55 °C, and 45 s at 72 °C, and a final extension time of 5 min at 72 °C.

### Immunohistochemical detection of intestinal HO-1

The paraffin-embedded intestine tissue was sliced into 5-μm sections and deparaffinized, followed by 30-min antigen retrieval using microwave treatment in 0.01 M citrate buffer (pH 6.0). Tissue sections were then incubated with 3% H_2_O_2_ for 20 min at room temperature and blocked in goat serum for 30 min at 37 °C. Afterwards, the sections were incubated with the primary antibody against HO-1 (Abcam, UK) at 4 °C overnight. The following day, tissue sections were incubated with the secondary antibody at room temperature for 1 h, using goat anti-rabbit IgG (Dako, Japan) according to the manufacturer’s instruction. Tissue sections were then stained with 3,3′-diaminobenzidine and counterstained with hematoxylin.

### Histological analysis

Intestinal tissues were processed by fixation in 10% formalin, paraffin embedded, and then sliced into 5-μm sections which were then stained with hematoxylin and eosin (H&E). Slides were blindly examined by light microscopy. Acute rejection was graded as described previously [35].

### Apoptosis assay

TdT-mediated dUTP-X nick end-labeling (TUNEL) staining was performed and apoptosis-positive cells were enumerated as described previously [34].

### Natural killer cell activity

Host natural killer (NK) cell function was evaluated by measuring lactate dehydrogenase (LDH) release following coculture with the target YAC-1 cells as described in our previous study [34]. Briefly, splenocytes and YAC-1 cells were cocultured, and the optical density at 570 nm was determined using an enzyme-linked immunosorbent assay (ELISA) microplate reader (BioTek Winooski VT 05404, USA) to calculate the level of NK cell activity.

### Measurement cytokines concentrations by ELISA

Recipient serum samples were collected from peripheral blood at each time point and stored at –80 °C for measurement. Interleukin (IL)-10, TGF-β, IL-2, IL-6, IL-17, IL-23, tumor necrosis factor (TNF)-α, and interferon (IFN)-γ concentrations were measured by ELISA kits as described by the manufacturer (BioSource, USA) and compared with standard curves.

### Detection of regulatory T cells by flow cytometry

Regulatory T (Treg) cells were stained and analyzed by flow cytometry as described previously [34]. Briefly, lymphocytes were isolated from the recipient spleens and incubated with antibodies against CD4-FITC, CD25-PE, and Foxp3-PerCP-Cyanine5.5 (eBioscience, San Diego, CA, USA). The CD4^+^CD25^+^FoxP3^+^ cells were analyzed by flow cytometry (FACSCalibur; BD).

### Statistical analysis

Results are expressed as mean ± SD and were compared by one-way analysis of variance. All statistical analyses were performed using SPSS statistical software, v. 17.0 (SPSS GmbH, Munich, Germany), and *P* ≤ 0.05 was used as a threshold to detect statistically significant differences.

## Results

### Characterization of Ad/HO-1/BMMSCs

**Fig. 1:**
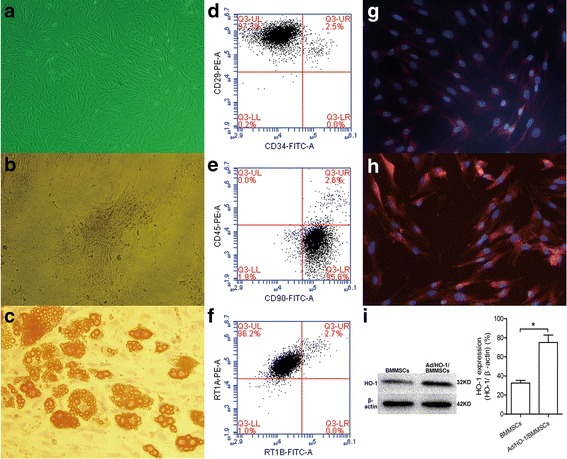
Characterization of Ad/HO-1/BMMSCs in vitro and detection of their HO-1 expression. (**a**) Morphology of Ad/HO-1/BMMSCs with a bright field. BMMSCs exhibit spindle-shaped morphology and are arranged in whorls when transduced with Ad/HO-1. (**b**) Osteogenic differentiation of Ad/HO-1/BMMSCs in vitro by Von Kossa staining. Black deposition of calcium salt can be seen in positive cells. (**c**) Adipogenic differentiation of Ad/HO-1/BMMSCs in vitro by Oil Red O staining. Red lipid droplet formation can be seen in positive cells. Surface markers detection of Ad/HO-1/BMMSCs demonstrated that the proportion of CD29-positive and CD34-negative cells was >97% (**d**), the proportion of CD90-positive and CD45-negative cells was >95% (**e**), and the proportion of RT1A-positive and RT1B-negative cells was >96% (**f**). Immunocytochemical staining revealed that the red fluorescence intensity (HO-1 expression level) in the cytoplasm was higher in the Ad/HO-1/BMMSC-treated group (**h**) than in the BMMSC-treated group (**g**). (**i**) Western blot analysis results showed significantly higher HO-1 expression in Ad/HO-1/BMMSCs than in BMMSCs (*p < 0.05). *Ad/HO-1* adenoviral vector carrying the heme oxygenase gene, *BMMSC* bone marrow mesenchymal stem cell, *HO-1* heme oxygenase-1

In accordance with our previous study [33, 34], BMMSCs isolated from rat bone marrow were spindle-shaped in morphology and had abilities of adherence to plastic, expressing specific surface molecules and multipotential differentiation in vitro (data not shown). BMMSCs transduced with recombinant adenovirus did not change in terms of cellular morphology (Fig. 1a) and were confirmed as BMMSCs based on their abilities for osteogenic differentiation and adipogenic differentiation in vitro (Fig. 1b, c). Ad/HO-1/BMMSCs were also identified through detection of cell surface molecules. Flow cytometry showed that over 95% of these cells were positive for CD29, CD90, and RT1A and were negative for CD34, CD45, and RT1B (Fig. 1d–f). The results demonstrated that transduction with recombinant adenovirus did not affect the molecular biological characteristics of BMMSCs.

### HO-1 transduction improved HO-1 expression in BMMSCs

The immunohistochemical staining results revealed that red fluorescence was visible with Ad/BMMSCs (Fig. 1g) and Ad/HO-1/BMMSCs (Fig. 1h). Specifically, the fluorescence intensity was higher in Ad/HO-1/BMMSCs than that in Ad/BMMSCs, especially 48 h after transduction. Western blot analysis results showed significantly higher HO-1 expression in Ad/HO-1/BMMSCs than in BMMSCs (Fig. 1i). The results indicated that the HO-1 gene was successfully overexpressed when transduced into BMMSCs.

### Improvement of recipient survival rates

**Fig. 2:**
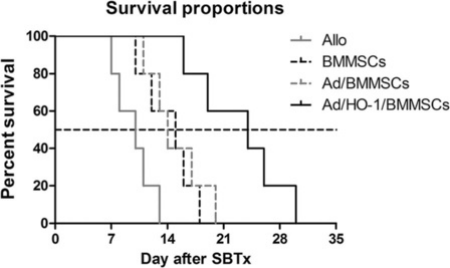
Kaplan–Meier survival curves of SBTx recipients. Median recipient survival times were 10, 15, 14, and 24 days, in the allogeneic control (*Allo*) , BMMSC-treated, Ad/BMMSC-treated, and Ad/HO-1/BMMSC-treated groups, respectively (*n* = 5 in each group). Survival rates were significantly improved in the Ad/HO-1/BMMSC treatment group compared with the control groups (*P* = 0.0018 vs allogeneic control group; *P* = 0.0088 vs BMMSC-treated group; *P* = 0.0333 vs Ad/BMMSC-treated group). *Ad* adenoviral vector, *BMMSC* bone marrow mesenchymal stem cell, *HO-1* heme oxygenase-1, *SBTx* small bowel transplantation

The median recipient survival times were 10, 15, 14, and 24 days in the allogeneic control group, BMMSC-treated group, Ad/BMMSC-treated group, and Ad/HO-1/BMMSC-treated group, respectively (Fig. 2). These results clearly demonstrate a significant improvement in survival rates in the Ad/HO-1/BMMSC group compared with the other control groups.

### Improvement of clinical manifestation and weight loss

**Fig. 3:**
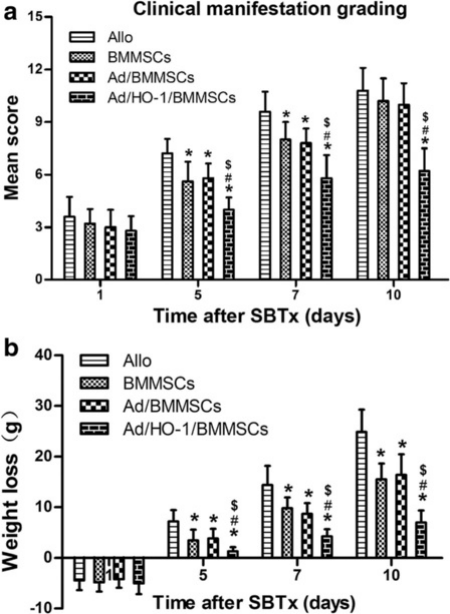
Clinical manifestation grading and weight loss of the recipients following SBTx. **a** Clinical manifestation scores were much higher in the allogeneic control (*Allo*) group compared with the BMMSC treatment group. HO-1 transduction markedly reduced the score, especially on days 7 and 10. **b** The majority of the animals suffered weight loss following SBTx. BMMSC treatment, especially when transduced with Ad/HO-1, significantly improved or even reversed this effect compared with the allogeneic control group. The least amount of weight loss occurred in the Ad/HO-1/BMMSC group, especially on days 7 and 10. Data expressed as mean ± SD (*P < 0.05 compared with allogeneic controls; ^#^ *P* < 0.05 compared with BMMSC controls; ^$^ *P* < 0.05 compared with Ad/BMMSC controls). *Ad* adenoviral vector, *BMMSC* bone marrow mesenchymal stem cell, *HO-1* heme oxygenase-1, *SBTx* small bowel transplantation

Quality of life became worse with time in the allogeneic control group. Clinical manifestations included asitia, weight loss, less activity, nonresponsiveness to stimulation, somnolence, and rat fur appearing scruffy and dull. These symptoms improved and the recipients were more active and dynamic in the treatment groups, especially in the Ad/HO-1/BMMSC group. Clinical manifestation grading results showed markedly reduced scores in the Ad/HO-1/BMMSC group compared with the controls (Fig. 3a). Weight loss became more apparent over time in each group as the dying animals were thin and bony by gross observation (Fig. 3b). However, the weight loss significantly improved with BMMSC treatment, especially when transduced with Ad/HO-1, as a comparison with the allogeneic control group. We found the lowest amount of weight loss in the Ad/HO-1/BMMSC group, especially on days 7 and 10. The results indicated that the general condition was better following Ad/HO-1/BMMSC treatment than in controls.

### Improvement of HO-1 protein and mRNA expression in small bowel graft by Ad/HO-1/BMMSC infusion

**Fig. 4:**
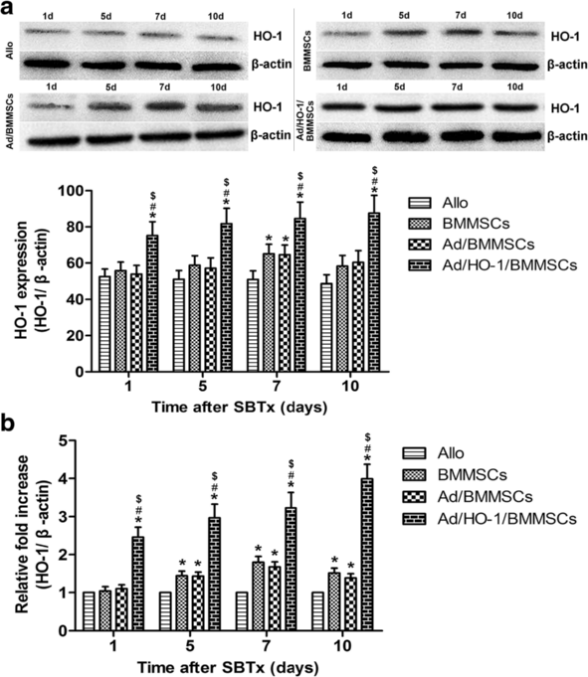
Expression of HO-1 protein and mRNA in the graft after SBTx. Expression of HO-1 in protein (**a**) and mRNA (**b**) was confirmed by western blot assay and real-time PCR, respectively (*n* = 5 in each group). Relative content was determined by HO-1/β-actin ratio. High-level and steady expression of HO-1 protein and mRNA were observed in the Ad/HO-1/BMMSC group (*P < 0.05 compared with allogeneic controls; ^#^ *P* < 0.05 compared with BMMSC controls; ^$^ *P* < 0.05 compared with Ad/BMMSC controls). *Ad* adenoviral vector, *Allo* allogeneic control, *BMMSC* bone marrow mesenchymal stem cell, *HO-1* heme oxygenase-1, *SBTx* small bowel transplantation

**Fig. 5:**
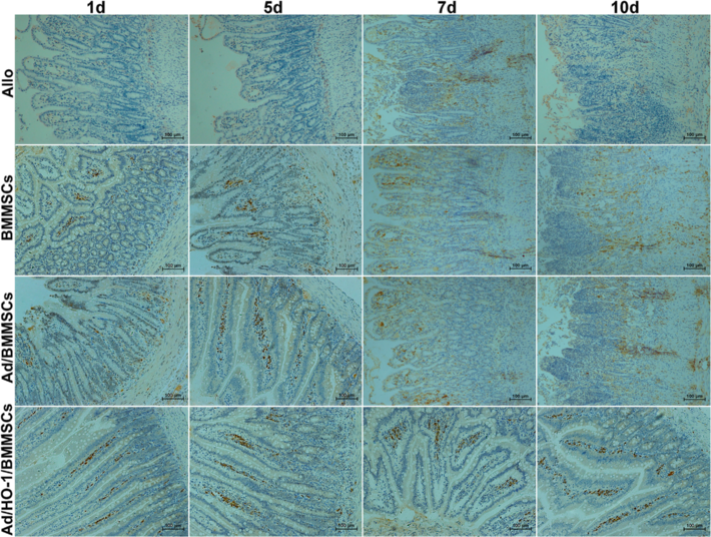
Immunohistochemical staining exhibiting HO-1 expression. HO-1 was detected primarily in the lamina propria of the small intestine villi and stained brown. In the allogeneic control (*Allo*) group, HO-1 was weakly expressed and difficult to detect. Both the BMMSC and the Ad/BMMSC treatment groups exhibited limited brown staining, while a greater amount of brown staining was detected in the Ad/HO-1/BMMSC treatment group. *Ad* adenoviral vector, *BMMSC* bone marrow mesenchymal stem cell, *HO-1* heme oxygenase-1

Western blot analysis results showed minute levels of HO-1 protein expression in the allogeneic control group. Both the BMMSC-treated and the Ad/BMMSC-treated groups exhibited low-level and downward expression of HO-1 protein. However, the Ad/HO-1/BMMSC treatment group demonstrated high-level and steady expression of HO-1 protein. PCR results showed that HO-1 mRNA expression increased less than 2-fold in the BMMSC-treated and the Ad/BMMSC-treated groups, and increased between 2.5-fold and 4-fold in the Ad/HO-1/BMMSC treatment group. Importantly, transduction with Ad/HO-1 resulted in a significant and steady increase in HO-1 gene expression compared with the control groups (Fig. 4). Immunohistochemical staining showed that HO-1 (stained brown) was expressed mainly by infused cells that localized in the lamina propria of the small intestine villi. Similar to the results of the western blot analysis, HO-1 was weakly detected in the allogeneic control group. Both the BMMSC-treated and the Ad/BMMSC-treated groups exhibited limited brown staining, while a greater amount of brown staining was detected in the Ad/HO-1/BMMSC treatment group (Fig. 5). These results suggest that BMMSCs may localize in the small intestine graft and function through HO-1 upregulation. HO-1 transduction could improve BMMSC survival and expression of HO-1 in small intestine graft.

### Improvement of small bowel graft histopathology and grading of acute rejection by Ad/HO-1/BMMSC infusion

**Fig. 6:**
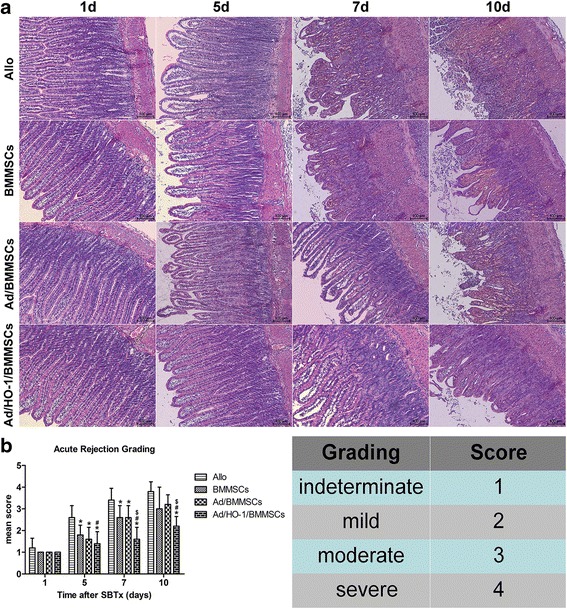
Histological sections of the rat small bowel and grading of ACR after SBTx. **a** Histological sections showing the pathologic changes in the small bowel in each group (H&E staining; magnification × 100). **b** Histogram of acute rejection grading (*n* = 5 in each group; *P < 0.05 compared with allogeneic controls; ^#^ *P* < 0.05 compared with BMMSC controls; ^$^ *P* < 0.05 compared with Ad/BMMSC controls). *Ad* adenoviral vector, *Allo* allogeneic control, *BMMSC* bone marrow mesenchymal stem cell, *HO-1* heme oxygenase-1, *SBTx* small bowel transplantation

Acute cellular rejection (ACR) was graded as indeterminate, mild, moderate, or severe, depending on findings in the mucosa and submucosa, including cell infiltration, crypt epithelial injury, apoptotic bodies in crypts, architectural distortion, and mucosal ulceration as described previously [35]. Allogeneically transplanted animals treated with normal saline showed a gradual increase in acute rejection, with moderate or severe acute rejection observed on day 7. Animals in both the BMMSC treatment group and the Ad/BMMSC treatment group showed reduced ACR to different degrees. However, the protective effect receded markedly after day 7, especially on day 10. ACR was maximally suppressed in the Ad/HO-1/BMMSC-treated group and the effect remained powerful 7 days post SBTx, with minimal cellular infiltration into the muscle layer and mucosa, fewest apoptotic cells, and mildest crypt destruction, compared with single BMMSC treatment or Ad/BMMSC treatment (Fig. 6).

### Reduction of apoptosis in small intestine mucosa by Ad/HO-1/BMMSC infusion

**Fig. 7:**
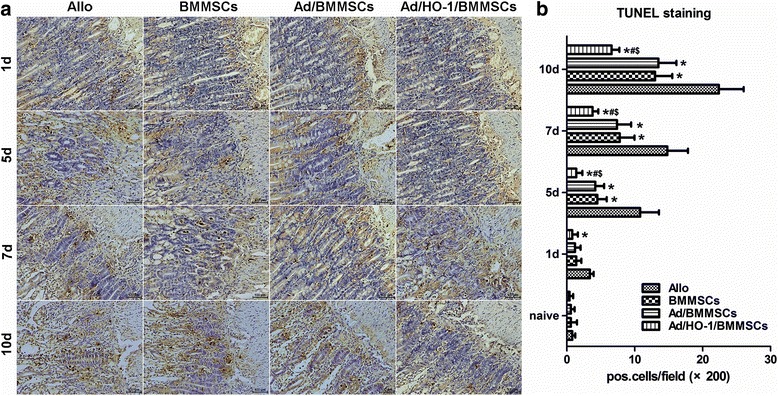
Apoptotic cells in graft mucosae. **a** Histological sections from each rat group at each time point were subjected to TUNEL staining (magnification × 200). **b** Histogram showing the percentage of positive apoptotic signals in each group on days 1, 5, 7, and 10 (*n* = 5 in each group). Apoptotic cells in graft mucosae increased rapidly in the allogeneic control (*Allo*) group; the increase was significantly inhibited by both BMMSC and Ad/BMMSC treatment. TUNEL-positive apoptotic cells further decreased in the Ad/HO-1/BMMSC treatment group compared with the treatment control groups. Data expressed as mean ± SD (*P* < 0.05 compared with the allogeneic controls; ^#^ *P* < 0.05 compared with the BMMSC controls; ^$^ *P* < 0.05 compared with the Ad/BMMSC controls). *Ad* adenoviral vector, *BMMSC* bone marrow mesenchymal stem cell, *HO-1* heme oxygenase-1, *TUNEL* terminal deoxynucleotidyl transferase-mediated dUTP nick end-labeling

Using TUNEL staining, we evaluated apoptotic cells in graft mucosa after SBTx (Fig. 7a). The numbers of apoptotic cells in the intestinal mucosa increased progressively in the normal saline allogeneic control group, and reduced to different degrees in both the BMMSC treatment group and the Ad/BMMSC treatment group. Compared with the other three control groups, TUNEL-positive apoptotic cells were least populated in the Ad/HO-1/BMMSC group (Fig. 7b); in particular, Ad/HO-1/BMMSC treatment significantly decreased cell apoptosis in the graft mucosa. Acute rejection is associated with increased apoptosis in the graft [36, 37]. The amount of apoptotic body in crypts was one of the criteria for diagnosing the grade of acute rejection in SBTx [35]. Our results indicated reduced acute rejection in all cell treatment groups, especially in the Ad/BMMSC treatment group.

### Reduction of recipient NK cell activity by Ad/HO-1/BMMSC infusion

**Fig. 8:**
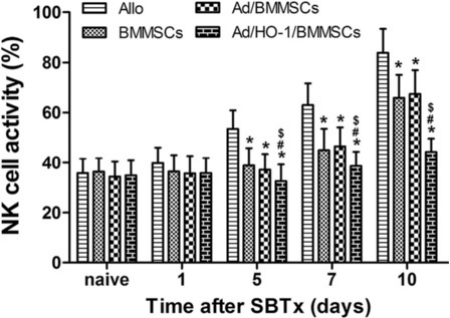
NK cell activity evaluated by measuring LDH release in mixed culture with YAC-1 cells. NK cell activity was significantly inhibited by BMMSC treatment or even dropped to the normal range when treated with Ad/HO-1/BMMSCs. Data expressed as mean ± SD (*n* = 5 in each group; *P < 0.05 compared with allogeneic controls; ^#^ *P* < 0.05 compared with BMMSC controls; ^$^ *P* < 0.05 compared with Ad/BMMSC controls). *Ad* adenoviral vector, *Allo* allogeneic control, *BMMSC* bone marrow mesenchymal stem cell, *HO-1* heme oxygenase-1, *NK* natural killer, *SBTx* small bowel transplantation

NK cell activity increased rapidly in the allogeneic control group, exceeding the normal range at all time points. While uptrend of NK cell activity was also presented in both the BMMSC treatment group and the Ad/BMMSC treatment group, the activity was significantly reduced compared with the allogeneic control group. Different to all three of these control groups, NK cell activity in the Ad/HO-1/BMMSC treatment group fluctuated within a small range and remained within the normal range at almost all time points. The results indicate that transduction with Ad/HO-1 successfully caused BMMSCs to be more capable of reducing NK cell activity after SBTx (Fig. 8). NK cell-mediated rejection in SBTx was thus most reduced in the Ad/HO-1/BMMSC treatment group, relative to the control groups.

### Recipient serum cytokine concentrations

**Fig. 9:**
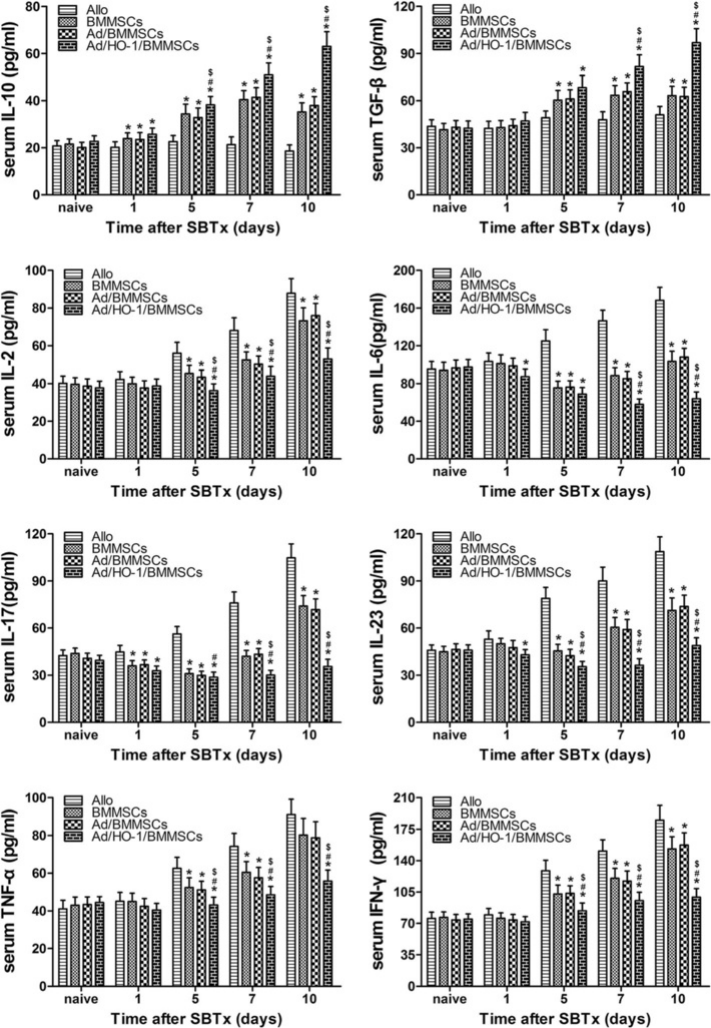
IL-10, TGF-β, IL-2, IL-6, IL-17, IL-23, TNF-α, and IFN-γ concentrations in recipient serum. ELISA revealed cytokine concentrations in the recipient serum on days 1, 5, 7, and 10 after transplantation (*n* = 5 in each group). Data expressed as mean ± SD (*P < 0.05 compared with allogeneic controls; ^#^ *P* < 0.05 compared with BMMSC controls; ^$^ *P* < 0.05 compared with Ad/BMMSC controls). *Ad* adenoviral vector, *Allo* allogeneic control, *BMMSC* bone marrow mesenchymal stem cell, *HO-1* heme oxygenase-1, *SBTx* small bowel transplantation

ELISA was performed to assay the serum concentrations of inflammatory response-related cytokines and T-cell differentiation-related cytokines such as Th1/Th2 and Th17/Treg (Fig. 9). In the allogeneic control group, the cytokine levels increased steadily after SBTx. Increases in IL-2, TNF-α, and IFN-γ levels were much lower in the BMMSC treatment group compared with the allogeneic control group. However, there was a sharp increase in their concentrations on day 10 compared with any other time points. Similarly, the Ad/BMMSC group demonstrated rising trends of IL-2, TNF-α, and IFN-γ levels between days 1, 5, 7, and 10, although these levels were significantly reduced compared with the allogeneic control group. Serum IL-10 and TGF-β concentrations in both the BMMSC treatment group and the Ad/BMMSC treatment group also increased rapidly between days 1, 5, and 7, and peaked on day 10. The IL-10 and TGF-β levels were significantly higher at each time point in these groups compared with the allogeneic control group. Moreover, IL-6, IL-17, and IL-23 concentrations in both the BMMSC group and the Ad/BMMSC group demonstrated a downward trend within 7 days, and an uptrend on day 10. These cytokine concentrations were significantly decreased compared with the allogeneic control group. As with the Ad/HO-1/BMMSC treatment group, concentrations of IL-2, TNF-α, and IFN-γ exhibited a relative small increase; levels of IL-6, IL-17, and IL-23 decreased further, while IL-10 and TGF-β concentrations increased continuously with respect to both the BMMSC and the Ad/BMMSC treatment groups. These results indicated that HO-1-transduced BMMSCs could maximally inhibit the inflammatory response, shift the Th1/Th2 balance to Th2, and differentiate into more Treg cells but fewer Th17 cells by maximally downregulating cytokines IL-2, IL-6, IL-17, IL-23, TNF-α, and IFN-γ, while upregulating cytokines IL-10 and TGF-β.

### Improvement of recipient spleen Treg cell levels by Ad/HO-1/BMMSC infusion

**Fig. 10:**
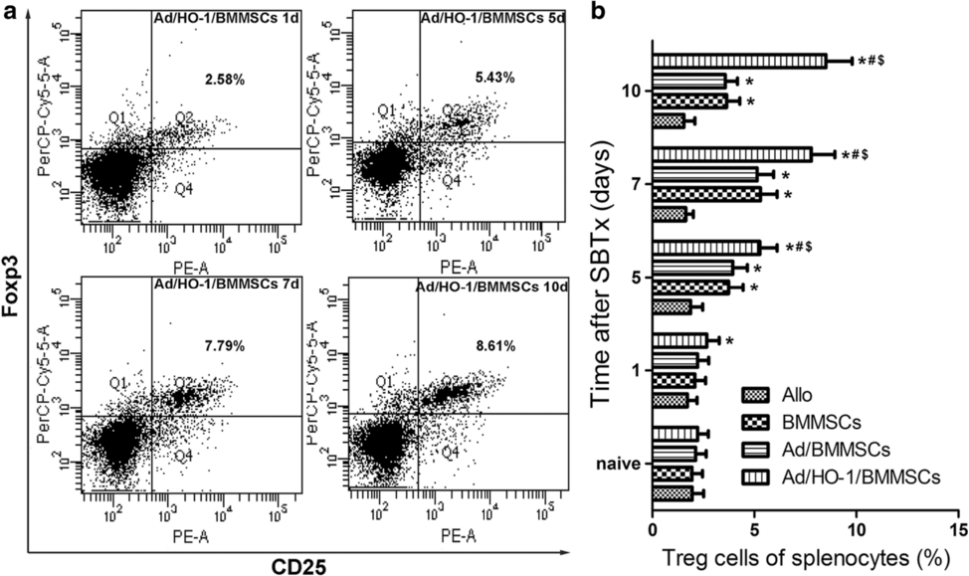
Expression of Treg cells in recipient spleen. **a** Scatter plot of Treg cells by flow cytometry. **b** Histogram showing the percentages of Treg cells on days 1, 5, 7, and 10 (*n* = 5 in each group). Levels of Treg cells increased steadily in the Ad/HO-1/BMMSC treatment group compared with the three control groups. Data expressed as mean ± SD (*P < 0.05 compared with allogeneic controls; ^#^ *P* < 0.05 compared with BMMSC controls; ^$^ *P* < 0.05 compared with Ad/BMMSC controls). *Ad* adenoviral vector, *Allo* allogeneic control, *BMMSC* bone marrow mesenchymal stem cell, *HO-1* heme oxygenase-1, *SBTx* small bowel transplantation

The numbers of CD4^+^CD25^+^Foxp3^+^ Treg cells in the spleens showed almost no change with time in the allogeneic control. In contrast, levels of Treg cells significantly increased in both the BMMSC treatment group and the Ad/BMMSC and Ad/HO-1/BMMSC treatment groups. In particular, a downward trend of Treg cells presented on day 10 in both the BMMSC and the Ad/BMMSC treatment groups, while a continuous increase was observed in the Ad/HO-1/BMMSC treatment group. In general, Treg cell levels were significantly increased in the Ad/HO-1/BMMSC treatment group (Fig. 10).

## Discussion

Although outcomes in SBTx have improved greatly in recent years, controlling graft rejection remains a big concern. Morbidity associated with acute and chronic rejection after SBTx has been reported as 90% and 30–50%, respectively [38]. Cell therapy, particularly in the form of MSCs, has emerged as a novel technique in suppressing the immune response after transplantation, owing to MSCs causing fewer side effects compared with immunosuppressants. While MSCs have been used in multifarious organ transplants [39–41] including SBTx [24, 42], there has been an increased focus on overcoming transplantation limitations and enhancing MSC function [43–47]. This study was designed to investigate whether the cytoprotective and immunosuppressive protein, HO-1, could improve the survival rate of BMMSCs in vivo and reinforce SBTx protection.

In this study, we first verified BMMSC transduction with Ad/HO-1 to make sure recombinant adenovirus transduction would not affect the BMMSCs, but mediated expression of the target gene. Cellular morphology and proliferation, in-vitro differentiation, and surface molecule characterization of our results all demonstrated that Ad/HO-1 did not alter the biological properties of BMMSCs, which was in agreement with previous studies [9, 43, 45]. Immunocytochemical staining revealed that the red fluorescence intensity in the cytoplasm was higher, while western blot analysis showed more HO-1 expression in Ad/HO-1/BMMSCs than in BMMSCs and in Ad/BMMSCs, indicating that the HO-1 gene was successfully overexpressed in the cytoplasm of Ad/HO-1/BMMSCs.

HO-1 is well known for its anti-inflammatory, anti-apoptosis, and cytoprotection properties. Recent studies have revealed its immunoregulatory role in MSCs, and particularly its immunosuppressive effect [31, 48]. Because we have demonstrated that HO-1 can be overexpressed when transduced into BMMSCs, we wondered whether overexpressed HO-1 in Ad/HO-1/BMMSCs could enhance their survival in a small bowel graft, and whether surviving cells expressed more HO-1. We therefore performed immunohistochemistry, western blot analysis, and PCR experiments to detect HO-1 expression in small bowel grafts. The results reveal a significant increase in expression of HO-1 protein and mRNA in the transplanted intestine in the Ad/HO-1/BMMSC treatment group. Importantly, HO-1 was mainly expressed by infused cells that localized in the lamina propria of the small intestine villi. In keeping with our conjecture, more BMMSCs survived in small bowel graft when transduced with Ad/HO-1.

To make clear whether improved survival of BMMSCs and the following HO-1 overexpression could be helpful to further reduce acute rejection, SBTx rejection was assessed histologically in a major histocompatibility complex (MHC)-disparate rat strain combination (BN to Lewis). Histological analysis showed that acute rejection gradually increased and reached moderate or severe levels on day 7 in the allogeneic control group, with massive destruction of crypt architecture, apoptosis, and heavy infiltration with neutrophils and lymphocytes. Both the BMMSC treatment group and the Ad/BMMSC treatment group presented alleviative acute rejection, with mild crypt distortion, apoptosis, and infiltration. In the Ad/HO-1/BMMSC treatment group, crypt distortion, apoptosis, and inflammatory cell infiltration were observed more rarely and the architectures were more integrated, indicating further attenuation of acute rejection. Grading of acute rejection significantly decreased in the Ad/HO-1/BMMSC treatment group, especially in the later stages, compared with the reduction in the BMMSC treatment group and the Ad/BMMSC treatment group. In addition, the recipient survival rate and quality of life factors such as weight loss significantly improved with alleviation of acute rejection.

The broad mechanisms of acute rejection in SBTx involve T-cell-mediated and antibody-based forms, which are similar to those in other organ systems. Fundamentally, the adaptive immune response, principally comprised of recipient-derived T-cell and B-cell subpopulations, has a complex interplay with innate immune populations including NK cells, dendritic cells, innate lymphoid cells, and macrophages [49]. Our study assessed several crucial aspects involving NK cell activity, T-cell subpopulations, and related cytokines to determine the effect of Ad/HO-1/BMMSCs on acute rejection.

NK cells mediate the non-MHC-restricted killing of virally infected and neoplastic cells and are involved in regulating the immune responses. Human NK cells mediate the rejection of both allogeneic bone marrow and xenogeneic solid organ grafts [50, 51]. We observed a significant increase in NK cell activity in the allogeneic control group. NK cell activity was reduced but still higher than normal in both the BMMSC treatment group and the Ad/BMMSC treatment group. Moreover, NK cell activity decreased to normal when treated with Ad/HO-1/BMMSCs.

Inflammation-related cytokines and T-cell differentiation-related cytokines play an important role in transplantation immunology. The Th1 cell subset mainly secretes IL-2, IFN-γ, and TNF-α, whereas Th2 cells mainly produce IL-4, IL-10, and IL-13. The Th1/Th2 paradigm and the cytokines related to these cells have been used to explain transplantation-related phenomena in immunity [52–54]. A previous study demonstrated that IL-2 was significantly elevated in acute rejection and correlated highly with the severity of rejection [55]. In contrast, high levels of IL-10 have been associated with the acceptance of solid allografts and even the induction of transplantation tolerance [56]. Furthermore, the IL-2/IL-10 ratio has been reported to be useful for the diagnosis of rejection in SBTx [57]. Also, TNF-α concentrations reportedly participate in acute rejection in organ transplantation [58–60]. The Th17/Treg axis switch serves a key function in regulating the immune responses in both organ transplantation and autoimmune disease [61, 62]. Therefore, we next assessed the regulation of Treg/Th17 according to their related cytokine secretion. Th17 cells are characterized by the production of IL-17, IL-21, and IL-22 [63, 64], and their differentiation requires TGF-β and IL-6 [65]. Stabilization of the Th17 cell phenotype is driven by IL-23, along with TNF-α and IL-1β [66]. Thus, we think measurement of these cytokine changes indirectly informs on T-cell polarization. We found that IL-10 and TGF-β were associated with Treg cells, while IL-6, IL-17, IL-23, TNF-α, and IFN-γ were associated with Th17. Our ELISA results revealed that the anti-inflammatory and Treg-associated cytokines, IL-10 and TGF-β, were significantly higher in the Ad/HO-1/BMMSC-treated group compared with the control groups. In contrast, the proinflammatory and Th17-associated cytokines IL-2, IL-6, IL-17, IL-23, TNF-α, and IFN-γ, reduced to different degrees in the treatment groups. It is worth noting that the effect was more powerful in the Ad/HO-1/BMMSC treatment group.

Treg cells are a subset of T cells that express Foxp3. Treg cells have anti-inflammatory functions and can promote tolerance to “self” by contact-mediated suppression or secretion of the anti-inflammatory cytokines IL-10 and TGF-β [67–71]. We demonstrated previously that expression of Treg cells is one of the mechanism by which BMMSCs display their immunosuppressive effect after SBTx [34]. Now, we reveal a further improvement in the levels of Treg cells in the Ad/HO-1/BMMSC treatment group compared with both the BMMSC and the Ad/BMMSC treatment groups.

## Conclusions

In summary, this study demonstrates that transduction with Ad/HO-1 significantly suppresses acute rejection after SBTx, improving both the survival rate of the SBTx recipient and the clinical outcomes of the procedure. We attribute this to the overexpression of HO-1, which enhances the immunosuppressive effect of BMMSCs through reduction of ACR, apoptosis, NK cell activity, and regulation of related cytokines and Treg cell expansion. Therefore, we conclude that HO-1 gene-modified BMMSCs play a reinforced effect in protecting the outcome of SBTx in rats.

## Abbreviations

ACR: Acute cellular rejection
Ad: Adenoviral vector
BMMSC: Bone marrow mesenchymal stem cell
BN: Brown Norway
DMEM: Dulbecco modified Eagle medium
ELISA: Enzyme-linked immunosorbent assay
HO-1: Heme oxygenase-1
IFN: Interferon
IL: Interleukin
NK: Natural killer
SBTx: Small bowel transplantation
TGF-β: transforming growth factor beta
TNF: tumor necrosis factor
Treg: Regulatory T
TUNEL: Terminal deoxynucleotidyl transferase-mediated dUTP nick end-labeling
